# Supplementary material for: Development of a novel mathematical model that explains SARS-CoV-2 infection dynamics in Caco-2 cells
Source: PeerJ. 2023 Feb 1;11:e14828. doi: 10.7717/peerj.14828 (PMC9899056; doi:10.7717/peerj.14828)
Supplement: Supplemental Information 1 [file peerj-11-14828-s001.pdf]

S1 APPENDIX: IMPLEMENTATION OF THE EVENTS IN THE DISCRETE MODEL

Assume that t is the time of the previous event, $t+\Delta_t$ is the time of the current event.

Type 1. In this case a cytokine enters a "regular" healthy cell, so the number of healthy cells U_0 is decreased by 1, the number of resistant cells U_1 is increased by 1 (we just change the type of the cell with index $U_0(t)$ from healthy to resistant, so implementation complexity is constant), and the number of cytokines Cyt is decreased by 1.

Type 2. In this case a virion enters a "regular" healthy cell, so the number of healthy cells U_0 is decreased by 1, the number of productive infected cells I^a is increased by 1 (we just swap types of several cells in the array: the cell number $U_0(t)$ is made resistant, the cell number $U_0(t)+U_1(t)$ is made infected with parameters $t^{inf}=t+\Delta_t$, $r=1$, $l=L_0$, so implementation complexity is constant), and the number of virions V is decreased by 1.

Type 3. In this case a virion enters a resistant cell, so the number of resistant cells U_1 is decreased by 1, the number of productive infected cells I^a is increased by 1 (we just make the cell number $U_0(t)+U_1(t)$ infected with parameters $t^{inf}=t+\Delta_t$, $r=1$, $l=L_0$, so implementation complexity is constant), and the number of virions V is decreased by 1.

Type 4. In this case a free virion enters an infected cell, so we select a random infected cell (let the index of this cell be equal to j, increment the value of the parameter r of the jth by 1 and decrease the number of virions V by 1, so the complexity of implementation of this event is also constant).

Type 5. In this case we perform a global update of the parameters of the continuous part of the model, i.e. the total numbers of free virions and cytokines, and local parameters of all infected cells. Let t_{prev} be the time of the previous execution of an event of the type 5 ($t_{prev}=0$ for the first occurrence), $\Delta=t+\Delta_t-t_{prev}$. For every infected cell we update the number of viral RNA using the formula

$$r=2^{\left(t+\Delta_t-t^{inf}\right)/\Delta_{t\,RNA\,double}}$$

(i.e. we assume that concentration of viral RNA grows exponentially until it reaches some predefined threshold). For every infected cell such that $t+\Delta_t-t^{inf}>\Delta_{t\,cyt}$ we evaluate the number of cytokines produced and add this number to Cyt

$$Cyt=Cyt+\min\{\Delta,t+\Delta_t-t^{inf}-\Delta_{t\,cyt}\}\cdot p_c$$

(i.e. we assume that cytokines are produced with a constant rate, and production starts after some latent period). For every productive infected cell such that $t+\Delta_t-t^{inf}>\Delta_{t\,latent}$ we evaluate the number of virions produced (taking into account resources utilized) and add this number to V:

$$\begin{aligned}
&\Delta_V=\min\left(\min\{\Delta,t+\Delta_t-t^{inf}-\Delta_{t\,latent}\}\cdot r\cdot l\cdot p_{Vir},l/n_{l2V}\right)\\
&V=V+\Delta_V\\
&l=l-\Delta_V\cdot n_{l2V}.
\end{aligned}$$

If resource concentration l drops to zero, the cell stops producing virions and becomes exhausted (similarly to the previous event types this operation is reduced to swapping some elements of the array and thus has a constant complexity).

Total complexity of implementation of this event is linear in the number of cells.

S2 APPENDIX: DETAILS OF THE CONTINUOUS MODEL

The function p is evaluated in the following way.

1. Consider the grid generated by the step Δ_h on the segment $[0,T]$;
2. For every i, $0\le i\le T/\Delta_h$, compute the number of virions $\varphi(i)$ produced up to the moment $i\cdot\Delta_h$ by a cell infected at the moment $t=0$. Evaluation of $\varphi(i)$ is based on the values $l(i)$ (concentration of resources such as lipids in the cell) and $r(i)$ (concentration of viral RNA in the cell). Initially $l(0)=L_0$, $r(0)=1$, $\varphi(0)=0$. After that we iteratively evaluate new values:
   - $r(i)=2^{i\Delta_h/\Delta_{t\,RNA\,double}}$;

- if $i\Delta_h < \Delta_{t\,latent}$, then $\Delta_V = 0$, else $\Delta_V = \Delta_h \cdot r(i) \cdot l(i) \cdot p_{Vir}$;
- $\varphi(i) = \varphi(i-1) + \Delta_V$;
- $l(i) = \max\{0, l(i-1) - \Delta_V n_{l2V}\}$.

The values computed are stored in memory.

3. Use linear interpolation to evaluate $p(t)$ taking into consideration the relation $p((i+1/2)\Delta_h) = (\varphi(i+1) - \varphi(i))/\Delta_h$.

Note that at the stage of equation solution evaluation of $p(t)$ requires constant complexity.

The system is solved numerically using a semi-implicit Euler method (see e.g. (Atkinson, 1989, p. 342)) with the fixed step Δ_{DE}. At the initial moment ($t = 0$) we set $U_0 = C_{initial}$, $U_1 = 0$, $C = 0$, $I = 0$, $V = V_{initial}$, $R = 0$. At the time of washing ($t = \Delta_{t\,clean}$) we set $V = Cyt = 0$. Note that in case of using a semi-implicit Euler method the sum of the first, the second and the fourth equation has zero in the right-hand side with some accuracy only.

S3 APPENDIX: GLOBAL AND LOCAL PARAMETER SELECTION PROCEDURE

Parameters were selected using the following implementation of coordinate descent method. Initial values were selected manually. The models were evaluated multiple times with various random parameters; we compared the resulting values of virions and RNA concentration with the ones obtained in the experiment and picked up the best fitting combination.

The step of the procedure is the following:

- compute Err for the original parameters;
- for every parameter P compute Err for $Params$ with the value of P multiplied by η_i,

$$\eta_i \in \left\{2^{-800}, 2^{-50}, 2^{-20}, 2^{-11}, 2^{4}, 2^{-2}, 2^{2}, 2^{4}, 2^{11}, 2^{20}, 2^{50}, 2^{800}\right\};$$

- select minimum of the error values computed; if it corresponds to the original set of values, stop; otherwise select the values corresponding to the minimum and go to the next step.

To speed the computations up all computed values are stored in memory, so for every combination of parameters considered evaluation of $Err()$ is performed just once, and then the required values are extracted from memory.

S4 APPENDIX: SIMPLE PROPERTIES OF THE SYSTEM IN THREE VARIABLES

Consider the system

$$\begin{cases} \dfrac{\mathrm{d}U}{\mathrm{d}t} = -\beta UV \\ \dfrac{\mathrm{d}I}{\mathrm{d}t} = \beta UV \\ \dfrac{\mathrm{d}V}{\mathrm{d}t} = pI \end{cases}$$

Without loss of generality assume that $U(0) = 1$, $I(0) = 0$, $V(0) = V_0 > 0$. In other words, $U(t)$ and $I(t)$ are the fractions of healthy cells and infected cells, respectively.

First show that in this model all cells get infected.

Assertion 1. $I(t) \to 1$ *as* $t \to \infty$.

Proof. The first two equations of the system imply that

$$\frac{\mathrm{d}U}{\mathrm{d}t} + \frac{\mathrm{d}U}{\mathrm{d}t} \equiv 0,$$

thus $U(t) + I(t) \equiv 1$. Obviously it holds that $I(t) \geq 0$, so the third equation implies that $V(t) \geq V_0$ for any $t \geq 0$. Thus

$$\frac{\mathrm{d}I}{\mathrm{d}t} = \beta UV = \beta(1-I)V \geq \beta V_0(1-I).$$

Hence it holds that

$$\frac{\mathrm{d}\ln(1-I)}{\mathrm{d}t} = -\frac{\mathrm{d}I/\mathrm{d}t}{1-I} \leq -\beta V_0.$$

As a result, if $t \to \infty$, then $\ln(1-I_0) - \beta V_0 t \to -\infty$ and $I \to 1$. □

Now show that the number of virions keeps growing linearly.

Assertion 2. *There exist $C_1, C_2 > 0$ and $t_0 \geq 0$ such that*

$$C_1 \cdot t \leqslant V(t) - V(t_0) \leqslant C_2 \cdot t$$

for all $t \geqslant t_0$.

Proof. By Assertion 1 there exists $t_0 > 0$ such that $I(t_0) > 1/2$. Obviously $U(t) > 0$ for all $t \geq 0$, so $I(t)$ is an increasing function and $I(t) \geq 1/2$ for all $t \geq t_0$. Thus is holds that

$$\frac{\mathrm{d}V}{\mathrm{d}t} = pI > p/2$$

and $V(t) \geqslant V(t_0) + p/2 \cdot t$ for all $t \geq t_0$. On the other hand obviously $I(t) \leq 1$ and $V(t) \leqslant V(t_0) + p \cdot t$. □

S5 APPENDIX: VERIFICATION OF PARAMETER INDEPENDENCE

In this section we provide mathematical background of parameter independence verification procedure.

Suppose that $x^{(0)} = \left(x_1^{(0)}, \ldots, x_n^{(0)}\right) \in \mathbb{R}^n$, $i_1, \ldots, i_k \in \{1, \ldots, n\}$. Denote the set

$$\left\{(x_1, \ldots, x_n) \in \mathbb{R}^n \quad | \quad x_l = x_l^{(0)} \text{ for } l \neq i_1, \ldots, i_k\right\}$$

by $\Pi^{i_1,\ldots,i_k}\left(x^{(0)}\right)$. Denote the set

$$\left\{x = (x_1, \ldots, x_n) \in \Pi^{i_1,\ldots,i_k}\left(x^{(0)}\right) \quad | \quad \left\|x - x^{(0)}\right\| = \varepsilon \text{ and } x_l = x_l^{(0)} \text{ for } l \neq i_1, \ldots, i_k\right\}$$

by $S_\varepsilon^{i_1,\ldots,i_k}\left(x^{(0)}\right)$ (here ε is some real-valued positive constant).

Definition 1. *Suppose that $F\colon \mathbb{R}^n \to \mathbb{R}$ is a continuous function that reaches a local minimum at a point $x^{(0)} = \left(x_1^{(0)}, \ldots, x_n^{(0)}\right)$. The set of coordinates $x_{i_1}, \ldots, x_{i_k}$ is said to be ε-dependent, if there exists a continuous curve γ parameterized by $t \in [0,1]$ such that $\gamma(t) \in \Pi^{i_1,\ldots,i_k}\left(x^{(0)}\right)$ for any $t \in [0,1]$, $\gamma(0) = x^{(0)}$, $\gamma(1) \in S_\varepsilon^{i_1,\ldots,i_k}\left(x^{(0)}\right)$ and $F(\gamma(t)) = F\left(x^{(0)}\right)$ for any $t \in [0,1]$. Otherwise the set $x_{i_1}, \ldots, x_{i_k}$ is referred to as ε-independent.*

Informally speaking, the curve γ specifies an implicit function that determines the dependence of the parameters $x_{i_1}, \ldots, x_{i_k}$. E.g., for the case of linear dependence it is sufficient to consider a linear combination of the respective parameters.

Assertion 3. *Suppose that a continuous function $F\colon \mathbb{R}^n \to \mathbb{R}$ achieves its local minimum F_0 at some point $x^{(0)}$ and there exists $\varepsilon > 0$ such that $F(x) > F\left(x^{(0)}\right)$ for any $x \in S_\varepsilon^{i_1,\ldots,i_k}\left(x^{(0)}\right)$. Then the set $x_{i_1}, \ldots, x_{i_k}$ is δ-independent for any $\delta > \varepsilon$.*

Proof. Assume that the set $x_{i_1}, \ldots, x_{i_k}$ is δ-dependent for some $\delta > \varepsilon$. Then by definition there exists a continuous curve γ parameterized by $t \in [0,1]$ such that $\gamma(t) \in \Pi^{i_1,\ldots,i_k}\left(x^{(0)}\right)$ for any $t \in [0,1]$, $\gamma(0) = x^{(0)}$, $\gamma(1) \in S_\delta^{i_1,\ldots,i_k}\left(x^{(0)}\right)$ and $F(\gamma(t)) = F\left(x^{(0)}\right)$ for any $t \in [0,1]$. Since $\delta > \varepsilon$, it holds that γ intersects $S_\varepsilon^{i_1,\ldots,i_k}\left(x^{(0)}\right)$ in some point $x' = \gamma(t')$. Thus the following chain of relations leads to a contradiction:

$$F_0 = F(\gamma(t')) = F(x') > F_0.$$

□

Assertion 3 implies the following procedure.

1. Consider a sufficiently small sphere around the minimum point obtained by optimization procedure.
2. Evaluate the value of the error function for a sufficiently dense mesh on this sphere and estimate the values outside the mash using some sort of interpolation.
3. If all values are greater than the minimum value, then there is no dependence outside the sphere, thus the parameters can be considered as independent.

REFERENCES

Atkinson, K. A. (1989). *An Introduction to Numerical Analysis*. John Wiley & Sons, New York, second edition.

SUPPORTING FIGURES

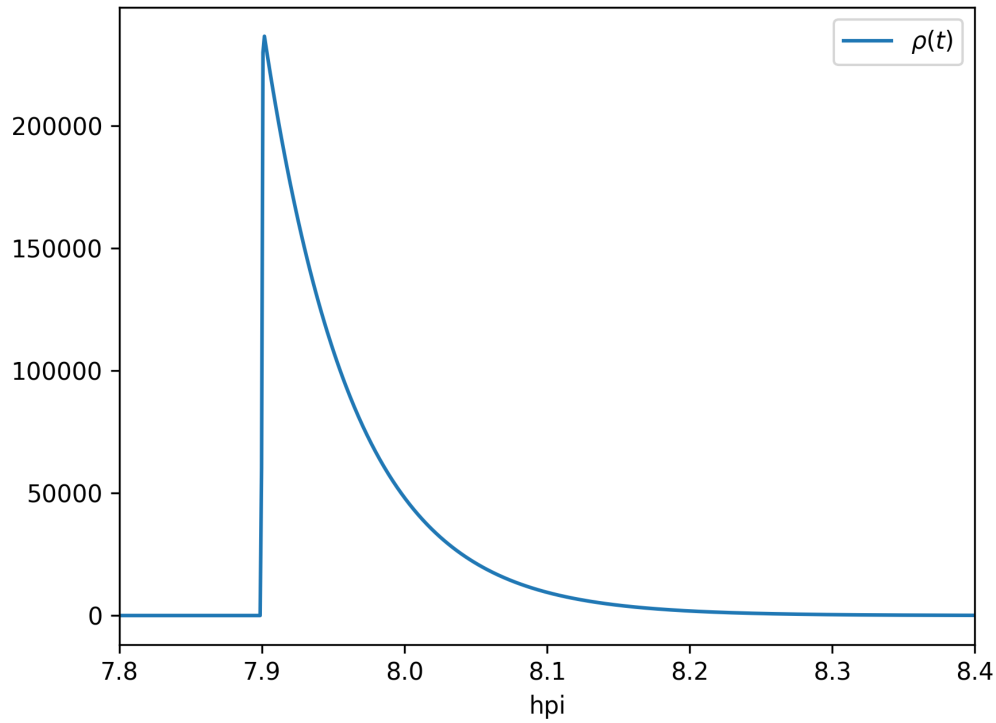

Figure S1. The plot of the function $p(t)$.

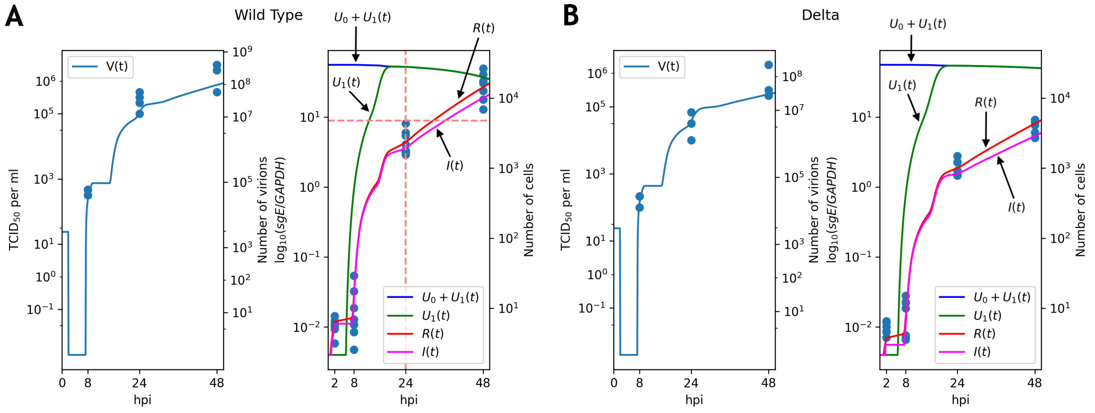

Figure S2. Solutions of the continuous model accurately explain experimental data for the WT (A) and Delta (B) variants. All parameters except two (cell entry rate β and cytokine production intensity p_c) are common for both variants. Dashed red lines stand for an experimental measurement of the percentage of WT-infected cells at 24 hpi.

SUPPORTING TABLES

	$\Delta_{t\,RNA\,double}$	k	p_c	β	β_{cyt}	$\Delta_{t\,latent}$	p_{Vir}	n_{l2V}
$\Delta_{t\,RNA\,double}$	—	0.564	0.518	0.960	0.512	0.988	0.388	1.62656
k	0.564	—	1.493	0.972	1.038	1.467	0.192	0.983
p_c	0.518	1.489	—	1.499	0.631	1.409	0.263	1.572
β	0.858	0.972	1.499	—	0.909	1.212	0.193	1.131
β_{cyt}	0.512	1.038	0.631	0.909	—	0.757	0.112	1.024
$\Delta_{t\,latent}$	1.764	1.467	1.409	1.212	0.757	—	0.892	1.285
p_{Vir}	0.313	0.192	0.263	0.193	0.112	0.168	—	0.294
n_{l2V}	0.681	0.983	1.572	1.131	1.024	1.285	0.294	—

Table S1. **Increase of the functional generated by pairs of parameters on a circle around the minimum point**
